# 

**Published:** 1896-08

**Authors:** 


### CONTENTS.

ORIGINAL COMMUNICATIONS—
"Skin Disease or 'Blood' Disease?" By M. B. Hutchins, M.D., Atlanta, Ga ....... 361
The Treatment of Hand Injuries. By William Perrin Nicolson, M.D., Atlanta, Ga. 366
One Hundred and Sixty six Cases of Cancer of the Pregnant Uterus Occurring Since 1886. By George H. Noble, M.D., Atlanta, Ga....... 375
Treatment of Abortion. By J. W. Long, M.D., ichmond, Va.... 386

SOCIETY REPORTS—
Clinical Society of Maryland.... 385
Richmond Academy of Medicine and Surgery.... 399

CORRESPONDENCE—
Medical New York.... 401
New York Letter.... 406

EDITORIAL—
The Tsetse Fly.... 413
Deaf-Mutes.... 415
Antitoxin.... 416
Substitution in Prescriptions.... 418
To New Subscribers.... 418

SELECTIONS AND ABSTRACTS—
Skin Diseases and Syphilis.... 419
General Medicine and Therapeutics.... 420
Miscellaneous.... 424

MEDICAL ITEMS.... 426
BOOK REVIEWS.... 430
MEDICAL ITEMS—Continued.... x, xi, xii, xiii
ADVERTISERS' INDEX TO ADVERTISEMENTS.... xiv
CLASSIFIED INDEX TO ADVERTISEMENTS.... xxxvii

